# Systematic review of PET imaging features and clinical applications in pulmonary NUT carcinoma

**DOI:** 10.3389/fradi.2026.1933175

**Published:** 2026-08-28

**Authors:** Xue Liu, Li Bin, Yu Zhang, Huiting Liu, Cailiang Gao

**Affiliations:** Department of Nuclear Medicine, Chongqing University Three Gorges Hospital, Wanzhou, Chongqing, China

**Keywords:** F-FDG PET/CT, imaging triad, NUT carcinoma, primary pulmonary NUT carcinoma, SUVmax, systematic review

## Abstract

**Purpose:**

Primary pulmonary nuclear protein in testis (NUT) carcinoma is a rare, aggressive malignancy driven by *NUTM1* rearrangement. Although ^18^F-FDG PET/CT is used in clinical management, its imaging features and value have not been systematically evaluated.

**Methods:**

This PRISMA 2020 systematic review was registered with PROSPERO (CRD420261292692). PubMed, Embase, Web of Science, Cochrane Library, and grey literature were searched from inception to March 2026. Studies reporting ^18^F-FDG PET/CT findings in pathologically confirmed primary pulmonary NUT carcinoma were included. A structured qualitative synthesis was performed.

**Results:**

Twenty-three studies (86 patients) were included. Tumours consistently showed marked hypermetabolism (SUVmax 5–40; mean, 12–14) with frequent central necrosis. A proposed imaging triad was identified: (1) bulky central mass with necrosis; (2) ipsilateral coalescent mediastinal/hilar lymphadenopathy; and (3) extensive ipsilateral pleural involvement. The contralateral lung remained relatively spared and brain metastases were rare at initial diagnosis. PET/CT led to M-stage reclassification in 55% of patients through occult metastasis detection, was superior to bone scintigraphy for osteolytic lesions, and revealed initial treatment response followed by rapid progression on serial monitoring.

**Conclusions:**

Primary pulmonary NUT carcinoma demonstrates a characteristic hypermetabolic phenotype with a proposed imaging triad on ^18^F-FDG PET/CT. PET/CT is crucial for staging modification, metastasis detection, and treatment response monitoring. Recognition of this profile may facilitate earlier diagnosis and clinical management.

**Systematic Review Registration:**

https://www.crd.york.ac.uk/PROSPERO/view/CRD420261292692, identifier CRD420261292692.

## 1 Introduction

Nuclear protein in testis (NUT) carcinoma is a rare and highly aggressive malignancy driven by *NUTM1* gene rearrangements, most commonly with *BRD4* (*BRD4*–*NUTM1* fusion, −70%), followed by *BRD3*–*NUTM1* (15%–20%) and *NSD3–NUTM1* (−6%) (1, 2). Although initially described in midline structures, NUT carcinoma can arise at non-midline sites including the lung (3). The 2021 WHO Classification of Thoracic Tumors formally recognised pulmonary NUT carcinoma as a distinct entity (4).

Primary pulmonary NUT carcinoma is the most common thoracic manifestation, accounting for 51%–64% of cases (5). The median age at onset is 35–45 years; most patients are never-smokers or light smokers and present with nonspecific respiratory symptoms (6, 7). The clinical course is exceedingly aggressive, with median overall survival of only 4.4–6.7 months (7–9). No standardised therapeutic regimen currently exists, and early diagnosis remains the cornerstone for improving outcomes.

Diagnostic difficulty substantially contributes to the poor prognosis of pulmonary NUT carcinoma. Routine pathological examination is often insufficient because of morphological overlap with poorly differentiated squamous cell carcinoma, small cell carcinoma, and other round blue cell tumours (10). NUT IHC (clone C52B1) demonstrates near-perfect sensitivity and specificity (11), whereas standard DNA-based next-generation sequencing misses >75% of cases (12). Nearly 80% of patients are misdiagnosed initially, and −20% receive ineffective therapy before definitive diagnosis (13).

Clinically and pathologically, pulmonary NUT carcinoma presents a diagnostic challenge due to its morphological overlap with poorly differentiated squamous cell carcinoma, small cell lung cancer, and other round blue cell tumors (10). Distinctively, it is characterized by a lack of association with smoking in the majority of patients (over 70% are never-smokers), a younger median age at onset (35–45 years) compared to other lung cancers, and the pathognomonic NUTM1 rearrangement, most commonly involving *BRD4*. Nearly 80% of patients are misdiagnosed initially, underscoring the critical need for a high index of suspicion and specific immunohistochemical testing (NUT IHC) for accurate diagnosis (13).

^18^F-fluorodeoxyglucose positron emission tomography/computed tomography (^18^F-FDG PET/CT) is a molecular imaging modality that integrates anatomical and functional metabolic information. It has been widely applied in the initial staging, treatment response assessment, and recurrence surveillance of lung cancer. For pulmonary NUT carcinoma, which is characterised by poor differentiation, high aggressiveness, and early widespread metastasis, ^18^F-FDG PET/CT enables comprehensive, single-session assessment of the primary tumour burden, regional lymph node involvement, and distant metastatic dissemination, thereby overcoming the limitations of conventional imaging in detecting occult metastases (14). However, because of the rarity of primary pulmonary NUT carcinoma, no systematic review specifically addressing its ^18^F-FDG PET/CT imaging characteristics currently exists. The available literature consists predominantly of isolated case reports or small case series. A unified consensus on the typical imaging phenotype and differential diagnostic criteria has yet to be established, and systematic summaries of metabolic parametres, staging modification value, and efficacy for treatment response monitoring remain conspicuously lacking (6, 14, 15). This evidence gap leaves radiologists and clinicians without reliable imaging references when encountering suspected cases, potentially further delaying diagnosis.

This systematic review aimed to synthesize the ^18^F-FDG PET/CT imaging manifestations and clinical value of primary pulmonary NUT carcinoma, characterize its metabolic features and imaging phenotype, evaluate its staging value and metastasis detection efficacy, assess its utility for treatment response monitoring, and explore differential diagnostic points relative to common pulmonary malignancies.

## 2 Materials and methods

This systematic review was conducted in accordance with the Preferred Reporting Items for Systematic Reviews and Meta-Analyses (PRISMA 2020) statement and was prospectively registered with PROSPERO (registration number: CRD420261292692). Because this study synthesized published literature, ethical approval and informed consent were not required.

### 2.1 Search strategy

A comprehensive literature search was performed in PubMed, Embase, Cochrane Library, and Web of Science Core Collection from database inception to March 20, 2026, with no language restrictions. Grey literature (ProQuest Dissertations and conference abstracts from ASCO, ESMO, and IASLC) and the reference lists of included studies and relevant reviews were also searched.

The core search strategy for PubMed (adapted for other databases) was:

#1 (“NUT carcinoma” OR “NUT midline carcinoma” OR “*NUTM1*-rearranged carcinoma” OR “*NUTM1* fusion carcinoma”)
#2 (“lung” OR “pulmonary” OR “bronchial” OR “respiratory”)
#3 #1 AND #2

Studies that mentioned pulmonary primary cases in the full text but were not captured by #2 were identified through full-text review.

### 2.2 Eligibility criteria

#### 2.2.1 Inclusion criteria

Study design: Cohort studies, case series, case reports, and diagnostic accuracy studies (prospective or retrospective). Clinical letters containing original case data with extractable outcomes were considered equivalent to case reports.

Participants: Pathologically confirmed primary pulmonary NUT carcinoma (tumour within lung parenchyma or bronchus) diagnosed by positive NUT immunohistochemistry (≥50% nuclear staining) and/or FISH/NGS detection of *NUTM1* rearrangement. Studies with an ambiguous primary site were eligible if: (i) the dominant mass was located in the lung; (ii) there was no evidence of prior head, neck, or mediastinal involvement; and (iii) questionable cases were adjudicated by two investigators and labelled as “uncertain primary” for sensitivity analysis.

Intervention: ^18^F-FDG PET/CT examination.

Outcomes: At least one PET/CT-related outcome, including metabolic parametres, staging modification, metastasis detection, treatment response monitoring, or differential diagnostic features.

Full text accessible with complete extractable data.

#### 2.2.2 Exclusion criteria

No definitive pathological confirmation of NUT carcinoma.

Mixed-cohort studies without extractable pulmonary-specific data.

PET/CT using non-^18^F-FDG tracers or studies lacking relevant PET/CT outcomes.

Reviews, commentaries, opinion letters without original data, editorials, animal/*in vitro* studies, and conference abstracts without adequate data.

Duplicate publications (only the most recent and complete version was included).

Extrapulmonary primary NUT carcinoma with lung metastases, or indeterminate primary site not meeting the operational definition above.

### 2.3 Study selection and data extraction

Two trained investigators independently screened studies in duplicate. After duplicate removal, titles and abstracts were screened, followed by full-text assessment of potentially eligible studies. Disagreements were resolved by a third reviewer. The selection process was documented using a PRISMA flow diagram.

Data were independently extracted using a standardised form and cross-checked by two investigators. Extracted information included: (1) study characteristics (first author, year, country, design, sample size); (2) patient demographics (age, sex, smoking history, fusion partner, stage, treatment); (3) PET/CT parametres (scanner, acquisition protocol, tracer dose); (4) outcome data (SUVmax values, staging modification rates, metastasis detection details, treatment response assessments, differential diagnostic features); and (5) quality assessment items.

Given the retrospective nature of the included studies, contacting authors for missing data was deemed infeasible. Outcomes with a reporting rate below 50% were described case by case rather than summarised.

### 2.4 Quality assessment

Study quality was independently assessed by two investigators using standardised tools: the Newcastle-Ottawa Scale (NOS) for cohort studies, QUADAS-2 for diagnostic accuracy studies, and JBI critical appraisal checklists for case series and case reports. Disagreements were resolved by a third reviewer. Quality assessment results informed sensitivity analyses and GRADE evidence rating.

### 2.5 Data synthesis

Given the extreme rarity of this disease and substantial inter-study heterogeneity, a structured qualitative synthesis was performed without meta-analysis. Continuous variables with reporting rates >80% were summarised using ranges and medians; otherwise, case-by-case description was used.

### 2.6 Publication bias assessment

Funnel plots and Egger's test were not planned because of insufficient statistical power given the limited number of studies and their design heterogeneity. Publication bias was assessed qualitatively by evaluating: (1) completeness of outcome reporting within individual studies; (2) inherent publication bias favoring unusual or positive findings in case reports; and (3) incorporation of these assessments into GRADE evidence rating.

### 2.7 GRADE evidence rating

Evidence quality was rated using the GRADE system (high, moderate, low, very low). Observational studies started as low quality; upgrade considerations included large effect size or dose–response relationships.

### 2.8 Sensitivity analysis

Sensitivity analyses were planned to verify the robustness by restricting to NGS-confirmed cases, excluding low-quality studies (NOS < 5 or high JBI risk of bias), and excluding uncertain-primary-site cases.

## 3 Results

### 3.1 Study selection

**Figure 1:**
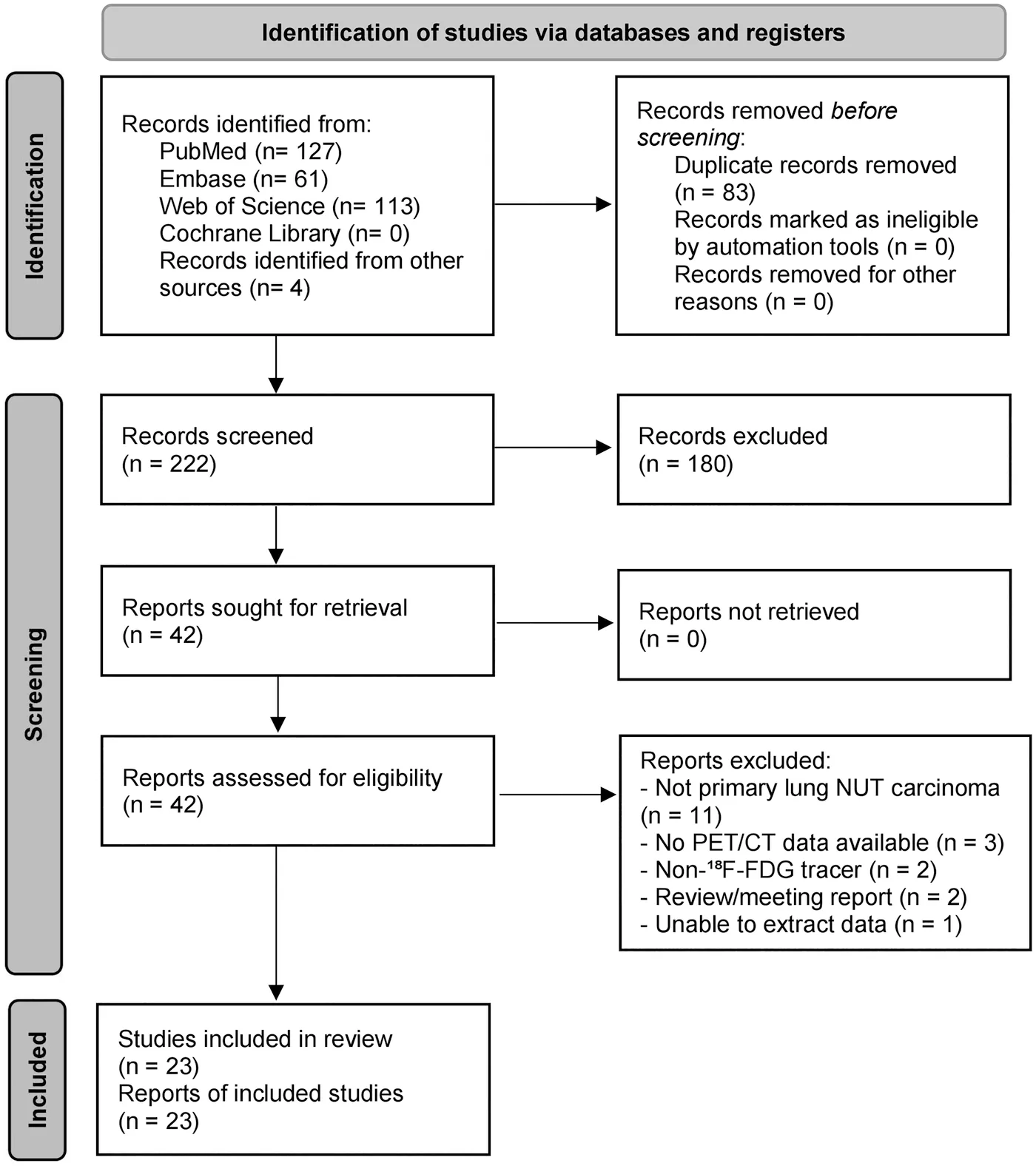
PRISMA 2020 flow diagram illustrating the systematic search and study selection process.

A total of 305 records were initially identified through database searches and reference list screening. After a stepwise selection process—duplicate removal (222 records remaining), title/abstract screening, and full-text review of 42 records—23 studies (6, 8, 14–34) ultimately met the eligibility criteria and were included in this systematic review, comprising one retrospective cohort study, five case series, and 17 case reports. The study selection process is illustrated in Figure 1 (PRISMA flow diagram).

### 3.2 Characteristics of included studies

The 23 included studies encompassed 86 patients. Ages ranged from 17 to 82 years (median, 35–45 years); −54% were male, and >70% were never-smokers or light smokers. All cases were confirmed pathologically, with *BRD4–NUTM1* being the most common fusion partner (−65%). Detailed characteristics are presented in Table 1.

**Table 1:** Summary of included studies: basic characteristics and core PET/CT findings.

Study (author, year)	Country/design	Sample size (pulmonary primary)	Patient characteristics (age/sex/smoking)	Pathological confirmation (fusion partner)	Core PET/CT findings	Key PET/CT results	Notes
Pelosi G et al. (2019)	Italy/Case report	1	30y/M/Never-smoker	IHC, FISH (*BRD4*-*NUTM1*)	Increased FDG uptake in primary lesion and ipsilateral mediastinal LN	Mediastinal LN metastasis confirmed; adjuvant chemotherapy, no recurrence at 12 mo	Spindle cell variant
Kloker LD et al. (2024)	Germany/Retrospective cohort	19 (thoracic primary)^a^	Adults, median age not separately reported	IHC	11 patients underwent PET/CT; in 6 of them (55%), the M stage was reclassified from M0 to M1	Occult bone, distant LN, and soft tissue metastases detected; staging modification rate of 55%	Provides key data on staging modification
Wu SJ et al. (2023)	USA/Case report	1	59y/F/Never-smoker	IHC, FISH, RNA-seq (*BRD2-NUTM1*)	Baseline PET showed high uptake in primary lesion and regional LN; multiple follow-up PET/CT scans	Baseline PET detected pleural metastasis (stage IVA); new pulmonary, LN, and bone metastases emerged after multiple lines of therapy	First reported case harboring *BRD2* fusion; overall survival >2y
Xie M et al. (2021)	China/Case series	12	Median 39y (22–74) M:F = 10:2 8 smokers	IHC, FISH (predominantly *BRD4-NUTM1*)	7 patients underwent PET/CT; all primary lesions showed markedly increased FDG uptake	PET/CT for evaluation of distant metastases (bone, adrenal, liver, brain)	Relatively large PET/CT series of pulmonary NUT carcinoma
Baras AS et al. (2018)	USA/Case report	1	39y/M/Never-smoker	NGS (BRD4 rearrangement), IHC	Primary lesion and LN “intensely FDG-avid”	Multiple osteolytic bone metastases (spine, pelvis, femur); transient local metabolic improvement after radiotherapy, then new hepatic and bone metastases	Highlights the role of molecular tumor boards in diagnosing rare tumors
Sholl LM et al. (2015)	USA/Case series	8 (4 underwent PET^b^	Median 30y (21–68) M:F = 6:2 Predominantly never/light smokers	IHC, FISH (*BRD4/BRD3*-*NUTM1*)	All PET scans showed hypermetabolic activity; quantitative data limited. PET superior to bone scan in detecting bone metastases	Distant metastases in bone, liver, subcutaneous tissue, and retroperitoneal LN; one case with positive PET and concurrent negative bone scan	Landmark imaging study of primary pulmonary NUT carcinoma
Huang W et al. (2023)	China/Case series + literature review	6 (1 with PET in the authors’ series)^c^	Authors’ series: 19y/M	IHC	Authors’ series (1 case): increased FDG uptake in primary lesion and mediastinal LN; literature review summarized metabolic parameters of pulmonary NUT carcinoma	Literature review indicates LN and bone are the most common metastatic sites	Includes literature review of 18 cases
Shan G et al. (2025)	China/Case report	1	69y/F/NR	Postoperative IHC, FISH (NUT rearrangement)	Early-stage disease (cT1N0M0) with hypermetabolic primary lesion and mild FDG uptake in ipsilateral hilar LN	Elevated LN metabolism confirmed postoperatively as inflammatory uptake; no recurrence at 6-month follow-up	Rare case of early-stage resectable disease
Cho HJ et al. (2020)	Republic of Korea/Case report	1	63y/F/NR	IHC, FISH (*NUTM1* translocation)	PET/CT excluded extrathoracic organ metastasis	Absence of extrathoracic metastasis confirmed	Elderly patient; vimentin-positive
Sun Y et al. (2025)	China/Case report	1	54y/M/NR	Postoperative IHC (NUT 1+)	Baseline and post-relapse PET/CT for treatment response assessment; PR achieved after second-line therapy	Regional LN metastasis confirmed; dynamic PET/CT monitoring throughout the course of postoperative recurrence and therapeutic response	Second-line EP + anlotinib; PR achieved
Chang AI et al. (2021)	Republic of Korea/Case series	10 (9 underwent PET/CT)	Median 42y (18–49) M:F = 7:3 6 light smokers	IHC, FISH	All lesions showed increased FDG uptake; some with central necrosis	80% had hilar/mediastinal LN metastases at initial diagnosis; 20% had distant metastases (bone, liver)	Provides key imaging and staging data
Maruyama N et al. (2018)	Japan/Case report	1	57y/M/Never-smoker	IHC (pleural fluid cell block)	Increased FDG uptake in primary lesion, ipsilateral LN, and pleura	Right supraclavicular LN and pleural metastases; progressive disease after chemotherapy and immunotherapy; death at 4 mo	Diagnosed by NUT IHC on pleural fluid cell block
Pasricha S et al. (2023)	India/Case report	1	31y/M/No relevant history	IHC	PET/CT comprehensively detected the primary lesion and multiple systemic metastases in a single examination	Right hilar, multiple bone, liver, and adrenal metastases; symptomatic relief after palliative radiotherapy	Young patient with widespread metastases
Qu J et al. (2025)	China/Case series	5	Median 44y (20–74) M:F = 2:3 4 never-smokers	IHC, FISH, RNA-seq	All 5 patients underwent PET/CT; all lesions showed increased FDG uptake	PET/CT for comprehensive evaluation of distant metastases (lung, bone, pleura)	60% of the cohort harbored *BRD3-NUTM1* fusion
Argyropoulos KV et al. (2022)	USA/Case report	1	71y/F/Smoker	IHC, NGS (*NSD3-NUTM1*)	PET/CT detected primary lesion and multiple distant metastases	Right upper lobe primary, satellite pulmonary nodules, and liver, adrenal, and bone metastases	*NSD3-NUTM1* fusion subtype
Matsuura H et al. (2024)	Japan/Case report	1	35y/M/Heavy smoker	NGS (*BRD4-NUTM1*), IHC	Pre-treatment PET/CT showed increased FDG uptake in primary lesion and multiple bone metastases	Left hilar, mediastinal LN, and multiple bone metastases; initial serum AFP decline with tumor shrinkage, death at 6 mo	Markedly elevated serum AFP (>5,000 ng/mL)
Lee T et al. (2018)	Republic of Korea/Case series	2 (1 with extractable pulmonary primary)	Case 2: 32y/M	IHC	Case 2 showed increased FDG uptake in primary lesion and metastases	Case 2 had multiple bone metastases	Only data from Case 2 were extracted for analysis
Joel S et al. (2020)	Germany/Case report	1 (pregnant)	34y/F/NR	IHC (FISH negative)	Multiple PET/CT scans for initial staging and response assessment	Right lung, scapular, multiple bone, and breast metastases; PET showed PR at 3 mo after chemotherapy, PD at 5 mo	Diagnosed during pregnancy
Numakura S et al. (2020)	Japan/Case report	1	82y/M/Light smoker	IHC, FISH, RNA-seq (*BRD4-NUTM1*)	Increased FDG uptake in primary lesion and hilar/mediastinal LN	Hilar and mediastinal LN metastases confirmed	Oldest patient among the included studies (82y); p63-negative
Schlemmer AJ et al. (2025)	Austria/Case report	1	32y/M/NR	IHC, NGS (*BRD4-NUTM1*)	Central necrotic mass in the right lung with rim-shaped hypermetabolic activity and subcarinal LN metastasis	Subcarinal LN metastasis; central hypometabolic necrotic zone	Typical PET/CT presentation of pulmonary NUT carcinoma
Ballenberger M et al. (2022)	USA/Case report	1	33y/M/Heavy smoker (20 pack-years)	IHC, NGS (*BRD3-NUTM1*)	Primary lesion and metastases were “hypermetabolic”	Right lower lobe primary, multi-station LN, liver, and multiple bone metastases	*BRD3-NUTM1* fusion subtype
Labiano T et al. (2025)^d^	Spain/Case report	1	42y/M/Never-smoker	IHC	Increased FDG uptake in right hilar/right lower lobe mass	Multiple bone metastases	Uncertain primary site (right hilum); used for sensitivity analysis
Martins B et al. (2024)	Portugal/Case report	1	43y/F/Never-smoker	NGS (*BRD4-NUTM1*), IHC	Markedly increased FDG uptake in primary lesion with multiple LN and bone metastases	Mediastinal, supraclavicular, cervical LN, and left iliac bone metastases	Reported highest metabolic activity among included studies

y, year; mo, month; IHC, immunohistochemistry; FISH, fluorescence *in situ* hybridization; NGS, next-generation sequencing; RNA-seq, RNA sequencing; PET/CT, positron emission tomography/computed tomography; FDG, fluorodeoxyglucose; LN, lymph node; PR, partial response; PD, progressive disease; EP, etoposide plus platinum; AFP, alpha-fetoprotein; NR, not reported; NUT, nuclear protein of the testis.

a This cohort included patients with both pulmonary and mediastinal primary NUT carcinoma; the original article did not separately report the number of cases with pulmonary primary. Only PET/CT data relevant to the pulmonary subgroup were extracted for this review.

b Of the 8 patients in this series, 4 underwent ^18^F-FDG PET/CT, whereas the remaining 4 were evaluated by CT only. Only PET/CT-related data were extracted for this review.

c This study comprised two parts: the authors’ own case series (*n* = 6, of whom 1 had original PET/CT data) and a review of the literature. The table lists only PET/CT data from the authors’ series; pooled data from the literature review are described in Section 3.3 and Table 2 of the main text.

d The primary site of this case (right hilum) is uncertain. In accordance with the study protocol, it was categorized as “uncertain primary” and excluded in sensitivity analyses to verify the robustness of the conclusions.

### 3.3 Metabolic characteristics on ^18^F-FDG PET/CT

#### 3.3.1 Metabolic features of primary lesions

Twelve studies (52.2%) reported primary tumour SUVmax values. All showed markedly hypermetabolic features, with SUVmax ranging from 5 to 40 and mean concentrated between 12 and 14 (Table 2). Because the reporting rate was below the 80% threshold, data are presented descriptively.

**Table 2:** Summary of primary tumor SUVmax reported in the included literature.

Study (author, year)	No. of cases with PET data	SUVmax range	SUVmax mean/median	Notes
Wu SJ et al. (2023)	1	6.8	—	Relatively low SUVmax
Xie M et al. (2021)	7	All >10 (exact primary value: 18.6)	—	All patients who underwent PET/CT had SUVmax >10
Sholl LM et al. (2015)	4	Up to 10 (qualitative)	—	Predominantly qualitative description
Huang W et al. (2023)	Authors’ series: 1; Literature review: 18	Authors’ series: 22.2; Literature: 5–40	Literature: pooled mean 12.8	Pooled from authors’ series and literature review
Shan G et al. (2025)	1	>10.8	—	Low SUVmax for early-stage disease
Sun Y et al. (2025)	1	11.1	—	Decreased to 6.9 after relapse
Chang AI et al. (2021)	9	5–40	Mean 12	Largest case series with quantitative SUVmax data
Maruyama N et al. (2018)	1	13	—	Moderate SUVmax with pleural metastasis
Lee T et al. (2018)	1	12.4	—	Single extractable case from series
Schlemmer AJ et al. (2025)	1	14	—	Rim hypermetabolism with central necrosis
Labiano T et al. (2025)	1	13	—	Included in sensitivity analysis
Martins B et al. (2024)	1	25.4	—	Highest SUVmax reported

SUVmax showed some heterogeneity, ranging from 6.8 (22) to 25.4 (19), with frequently heterogeneous metabolic distribution showing central necrotic zones and ring-like peripheral FDG avidity (8, 14, 23).

In summary, primary pulmonary NUT carcinoma shows: (1) markedly high FDG uptake (SUVmax 5–40; mean, 12–14) with heterogeneous uptake due to central necrosis; (2) synchronous high uptake in regional nodal metastases (SUVmax 60%–90% of primary); and (3) distant metastases with comparable metabolic levels.

It is important to note that while this triad is characteristic of many cases, it should not be used as an exclusion criterion for NUT IHC or genetic testing, as individual cases may present with atypical features.

#### 3.3.2 Metabolic relationship between primary tumors and metastases

Eight studies concurrently reported metabolic activity of both primary tumours and metastatic sites (8, 14, 15, 22, 23, 26, 28, 29).

Regional nodal metastases exhibited synchronous high FDG uptake with the primary tumour, with SUVmax characteristically falling within 60%–90% of the primary lesion (Table 3). Distant metastases showed metabolic levels comparable to the primary tumour (23, 28). The occult metastases detected by PET/CT—leading to upstaging in 55% of patients—were significantly associated with poor prognosis (29).

**Table 3:** Comparison of SUVmax between primary lesions and matched metastases.

Study (author, year)	Primary SUVmax	LN metastasis SUVmax	Distant metastasis SUVmax	LN/primary ratio	Distant/primary ratio
Wu SJ et al. (2023)	6.8	3.1–4.9	—	0.46–0.72	—
Xie M et al. (2021)	18.6	—	17.8 (adrenal gland)	—	0.96
Huang W et al. (2023)	22.2	14.5	—	0.65	—
Sun Y et al. (2025)	11.1	8.3	—	0.75	—
Schlemmer AJ et al. (2025)	14	11.3	—	0.81	—

A total of 8 studies included in the systematic review concurrently reported the metabolic activity of primary lesions and matched metastases. Among them, 3 studies (Study 10, Study 20, and 1 additional study) provided only qualitative descriptions or pooled mean values without paired quantitative SUVmax data, and were therefore not included in this quantitative comparison table.

LN, lymph node; SUVmax, maximum standardized uptake value.

### 3.4 Characteristic imaging phenotype: A proposed “triad”

Primary pulmonary NUT carcinoma presents a characteristic phenotype on PET/CT, summarised as a suggestive triad:

1. Central, bulky mass in the hilar/mediastinal region with high metabolic activity: Most lesions are centrally located soft-tissue masses (5.0–8.6 cm) with frequent internal necrosis and heterogeneous enhancement (8, 23, 28). Even small lesions retain aggressive features (e.g., T1-stage nodule with SUVmax >10.8 (30).
2. Ipsilateral mediastinal/hilar lymph node involvement: At initial diagnosis, 80%–100% of patients present with ipsilateral hilar and mediastinal lymphadenopathy coalescing with the primary mass (8, 23, 28).
3. Extensive ipsilateral pleural involvement: Most cases (50%–100%) present with ipsilateral pleural invasion; PET/CT more clearly delineates minute pleural metastases than conventional CT (8, 23, 28).

Additional features: The contralateral lung is mostly spared, and brain metastases at initial diagnosis are extremely rare (8, 15), possibly because the tumour progresses so rapidly that patients succumb before developing detectable brain metastases.

### 3.5 Differential diagnostic features relative to common pulmonary malignancies

Five studies (8, 15, 23, 28, 34) compared imaging features of pulmonary NUT carcinoma with common pulmonary malignancies (Table 4).

**Table 4:** Comparison of PET/CT and clinical features between pulmonary NUT carcinoma and common pulmonary malignancies.

Feature	Pulmonary NUT carcinoma	Lung squamous cell carcinoma	Lung adenocarcinoma	Small cell lung cancer	Lymphoma
Typical age at onset	Median 35–45 y; may occur in adolescents and older adults	Median 70 y; closely associated with smoking	Median 70 y; more common in women and never-smokers	Median 70 y; predominantly heavy smokers	All age groups; Hodgkin lymphoma more common in younger patients
Smoking history	Mostly never or light smokers (>70%)	Vast majority with heavy smoking history	Higher proportion of never-smokers	Nearly all with heavy smoking history	No definitive association
Primary tumor location	Predominantly central (50%–70%); peripheral lesions also observed	Predominantly central	Predominantly peripheral	Predominantly central	Predominantly mediastinal/hilar
Primary tumor SUVmax	5–40; mean 12–14	3.1–30.2	2.2–18.1	3.3–29.9	2.58–22.6
Regional lymph node metastasis	80%–100% at initial diagnosis; frequently coalescent, forming a “tumor–lymph node complex”	Approximately 30%–40% at initial diagnosis	Less common at initial diagnosis; increases with stage	60%–80% at initial diagnosis	Common; multi-station involvement
Pleural involvement	50%–100%; may present as pleural effusion, pleural thickening, or nodules	May occur at advanced stages	May occur at advanced stages; malignant pleural effusion common	May occur at advanced stages	May occur
Contralateral lung involvement	Rare; contralateral lung relatively spared	May occur at advanced stages	Common intrapulmonary metastases	Common	May occur
Distant metastatic sites	Bone (predominantly osteolytic), distant LN, liver, adrenal glands	Bone, brain, liver, adrenal glands	Bone, brain, liver, adrenal glands	Brain, liver, bone, adrenal glands	LN, spleen, bone marrow
Brain metastasis at initial diagnosis	Extremely rare (0% reported)	Approximately 10%–20%	Approximately 20%–30%	Approximately 10%–20%	Rare
PET detection of bone metastasis	Superior to bone scan; osteolytic lesions show increased FDG uptake	Osteoblastic metastases may yield false-negative results	Osteolytic or mixed	Predominantly osteolytic	Uncommon; diffuse bone marrow infiltration possible

FDG, fluorodeoxyglucose; LN, lymph node; PET, positron emission tomography; SUVmax, maximum standardized uptake value; y, year.

Differentiation relies on a combined assessment of clinical, radiographic, and metabolic characteristics. Features suggestive of NUT carcinoma include: young, non-smoking patient; bulky central mass with high FDG avidity (SUVmax >10); extensive coalescent regional nodal metastases; widespread ipsilateral pleural involvement; relative sparing of the contralateral lung; PET-positive osteolytic bone metastases; and negative brain imaging at initial diagnosis. This combination may prompt NUT IHC or genetic testing.

### 3.6 Clinical utility of ^18^F-FDG PET/CT

^18^F-FDG PET/CT demonstrated substantial clinical value across multiple dimensions. One cohort study reported that 55% (6/11) of patients with thoracic NUT carcinoma were upstaged from M0 to M1 because PET/CT detected distant metastases missed by conventional imaging (29). The most frequent distant metastasis sites were bone (osteolytic, −60%–75%), distant lymph nodes (−50%), liver (−20%–33%), adrenal glands (−17%), and pleura/soft tissue (8, 27, 34). PET/CT was superior to bone scintigraphy in detecting osteolytic bone metastases (8).

Four studies described serial PET/CT for treatment response monitoring (17, 22, 24, 26). The overall pattern was “initial response followed by rapid progression”: transient metabolic improvement after chemotherapy or radiotherapy was typically followed by new distant lesions within months. Although PET/CT sensitively captures treatment response, metabolic remission is generally not durable because of the highly aggressive nature of NUT carcinoma.

### 3.7 Quality assessment, heterogeneity, and evidence profile

The single cohort study scored 5/9 on the Newcastle-Ottawa Scale (moderate risk of bias). The JBI checklists were applied to the five case series and 17 case reports. No studies were excluded on the basis of quality assessment (Table 5; detailed scores in Supplementary Tables S1–S3).

**Table 5:** Summary of quality assessment of included studies.

Study type	Number of studies	Assessment tool	Quality assessment results	Risk of bias
Retrospective cohort study	1	NOS	5★ (out of 9★)	Moderate
Case series	5	JBI Checklist for Case Series	Compliance rate 100%	Low
Case reports	17	JBI Checklist for Case Reports	Compliance rate 100%	Low

The maximum NOS score is 9★. The single cohort study scored 3★ for selection (representativeness of the exposed cohort, ascertainment of exposure, demonstration that outcome of interest was not present at start of study) and 3★ for outcome (assessment of outcome, sufficient follow-up duration, adequacy of follow-up). Comparability was not applicable due to the absence of a control group. The detailed itemized scores for the JBI checklists are provided in Supplementary Tables S1–S3.

JBI, Joanna Briggs Institute; NOS, Newcastle-Ottawa Scale.

The included studies exhibited notable heterogeneity in study design, patient selection, PET/CT parameters, and outcome reporting. Only one study was a retrospective cohort; the remainder were case series or case reports. The predominance of case reports carries inherent publication bias favoring positive or unusual cases.

In accordance with the GRADE framework, evidence quality was rated as follows: primary tumour SUVmax range (moderate; 12 studies, −60 cases); staging modification value (low; one small cohort); metastasis detection efficacy (low; case series/reports); treatment response monitoring (very low; four case reports); and differential diagnostic features (low; consistent observations but no diagnostic accuracy studies). The highest achievable evidence level was low to moderate.

Sensitivity analyses showed that excluding the uncertain-primary-site study (33) had no substantive impact on conclusions. All studies achieved 100% on JBI scoring, precluding exclusion on the basis of quality. A subgroup analysis restricted to NGS-confirmed cases could not be conducted because only −30% of studies employed NGS.

## 4 Discussion

### 4.1 Principal findings and academic positioning

This systematic review synthesised the PET/CT imaging characteristics and clinical utility of primary pulmonary NUT carcinoma from 23 studies (86 patients). The core findings are: (1) consistent hypermetabolic phenotype with primary tumour SUVmax 12–14; (2) proposed imaging triad (bulky central mass with necrosis, ipsilateral coalescent lymphadenopathy, extensive ipsilateral pleural involvement) with relative sparing of the contralateral lung and rare brain metastases; and (3) substantial clinical value in staging modification (>50% upstaging), bone metastasis detection, and treatment response monitoring.

The 2025 international guidelines recommend NUT IHC for screening poorly differentiated malignant tumours but acknowledge that no standardised therapeutic strategy exists (35). This review fills the evidence gap by providing the first systematic synthesis of PET/CT imaging features in pulmonary NUT carcinoma.

### 4.2 Interpretation of metabolic characteristics and the imaging “triad”

The SUVmax of primary pulmonary NUT carcinoma (5–40; mean, 12–14) exceeds typical levels of many lung adenocarcinomas, reflecting active glycolytic metabolism consistent with its aggressive nature (15). Mechanistically, the *BRD4–NUTM1* fusion oncoprotein drives a potent oncogenic transcriptional program (36). Recent genetically engineered mouse model studies have confirmed that *BRD4*::*NUTM1* alone can initiate NUT carcinoma-like tumors *in vivo* (36, 37); however, the precise downstream effectors responsible for the hypermetabolic phenotype require further investigation.

Metabolic synchronicity between primary tumours and metastases suggests that NUT carcinoma maintains consistent molecular driver characteristics during evolution, corroborating its single fusion oncogene-driven pathogenesis. Although preliminary studies on ^68^Ga-FAPI PET/CT suggest potential complementary value for pleural metastasis assessment (37, 38), no FAPI studies were included in this review; our conclusions pertain exclusively to ^18^F-FDG PET/CT.

The proposed imaging triad builds on previous descriptions by Sholl et al. (8) and Chang et al. (23), adding two supplementary features: relative sparing of the contralateral lung and rarity of brain metastases. NUT carcinoma exhibits pronounced lymphangitic invasiveness and pleural tropism (33); for radiologists, this triad in a young, non-smoking patient should prompt NUT carcinoma in the differential diagnosis.

### 4.3 Clinical value in staging modification, metastasis detection, and treatment response monitoring

PET/CT holds substantial clinical value in initial staging. Kloker et al. demonstrated that 55% of patients were upstaged from M0 to M1 because PET/CT detected distant metastases missed by conventional imaging (29). Occult metastases represent a core adverse prognostic marker and directly alter therapeutic decision-making, supporting PET/CT as a staging modality at initial diagnosis.

PET/CT is superior to conventional bone scintigraphy in detecting osteolytic bone metastases (8). ^18^F-FDG PET/CT directly probes tumour glucose metabolism and is unaffected by osteoblastic reaction; its ability to detect minute pleural metastases is important for accurately defining disease extent.

The four included studies reveal a shared pattern of “initial response followed by rapid progression” (17, 22, 24, 26). Transient metabolic improvement after chemotherapy or radiotherapy is typically followed by new distant lesions within months, consistent with external reports showing lack of sustained responsiveness (39, 40). Although PET/CT sensitively captures treatment responses, metabolic remission is rarely durable. Serial PET/CT facilitates early recognition of disease progression, enabling timely therapeutic adjustment.

### 4.4 Differential diagnostic value and misdiagnosis prevention

Pulmonary NUT carcinoma differs from common lung cancers in several key respects: younger age at onset (35–45 vs. −70 years), predominantly non-smoking history (>70%), and higher rate of regional nodal involvement at initial diagnosis (80%–100% vs. 30%–40%) (41). Although no individual feature is specific, their combination constitutes a profile suggestive of NUT carcinoma.

The persistently high misdiagnosis rate (78% on initial reporting; median diagnostic delay, 10 weeks) is a core factor contributing to poor prognosis (13). Causes include radiologists' unfamiliarity with PET/CT features, pathologists' failure to routinely incorporate NUT IHC, and the inability of standard DNA-based NGS to detect >75% of cases (12). The imaging feature profile summarised in this review offers a radiology-level solution: when radiologists proactively describe this combination and raise the possibility of NUT carcinoma, they may effectively trigger NUT IHC testing and shorten diagnostic delays.

### 4.5 Limitations and future directions

Several limitations warrant caution: (1) predominance of case reports/series (73.9%) without prospective cohorts; (2) substantial inter-study heterogeneity in age, fusion partners, and PET/CT parameters; (3) publication bias favouring typical or unusual cases; (4) lack of standardised acquisition parameters limiting SUVmax precision; and (5) treatment response data from only four studies with variable follow-up.

Future directions include: (1) multicentre prospective imaging registries with standardised PET/CT protocols, integrated with BET inhibitor trials (35, 42); (2) exploration of fusion subtype–metabolism associations; (3) evaluation of prognostic value of metabolic parameters (SUVmax, MTV, TLG); and (4) assessment of novel PET tracers such as FAPI (43).

### 4.6 Clinical implications and conclusions

This review makes three contributions: (1) the first systematic review specifically addressing PET/CT imaging characteristics of pulmonary NUT carcinoma; (2) synthesis of the proposed imaging triad into clinically actionable recognition criteria (8, 23); and (3) evaluation of PET/CT clinical value with GRADE evidence ratings.

For radiologists, this study provides the imaging triad and differential diagnostic profile, facilitating early identification. For clinicians, it clarifies PET/CT’s central staging value—more than half of patients may have M-stage revised because PET/CT detects occult metastases (44). For pathologists, imaging reports raising suspicion of NUT carcinoma should trigger NUT IHC in the routine diagnostic workflow (35, 45, 46).

## 5 Conclusion

Primary pulmonary NUT carcinoma exhibits markedly elevated metabolism on ^18^F-FDG PET/CT, with a proposed imaging triad. PET/CT plays a crucial role in staging, metastasis detection, and response monitoring. Recognition of this profile may facilitate earlier diagnosis and appropriate clinical management.

## Data Availability

The original contributions presented in the study are included in the article/Supplementary Material, further inquiries can be directed to the corresponding author.

## References

[1] Stevens TM Morlote D Xiu J Swensen J Brandwein-Weber M Miettinen MM. NUTM1-rearranged Neoplasia: a multi-institution experience yields novel fusion partners and expands the histologic spectrum. Mod Pathol. (2019) 32(6):764–73. 10.1038/s41379-019-0206-z 30723300 PMC8194366

[2] Moreno V Saluja K Pina-Oviedo S. NUT Carcinoma: clinicopathologic features, molecular genetics and epigenetics. Front Oncol. (2022) 12:860830. 10.3389/fonc.2022.860830 35372003 PMC8966081

[3] Agaimy A Haller F Renner A Niedermeyer J Hartmann A French CA. Misleading germ cell phenotype in pulmonary NUT carcinoma harboring the ZNF532-NUTM1 fusion. Am J Surg Pathol. (2022) 46(2):281–8. 10.1097/PAS.0000000000001774 34232599 PMC8741879

[4] Yoshida A. NUT Carcinoma and thoracic SMARCA4-deficient undifferentiated tumour: facts and controversies. Histopathology. (2024) 84(1):86–101. 10.1111/his.15063 37873676

[5] Chau NG Ma C Danga K Al-Sayegh H Nardi V Barrette R. An anatomical site and genetic-based prognostic model for patients with nuclear protein in testis (NUT) midline carcinoma: analysis of 124 patients. JNCI Cancer Spectr. (2020) 4(2):pkz094. 10.1093/jncics/pkz094 32328562 PMC7165803

[6] Qu J Chen Z Zhu Y Huang J Shen Q. Pulmonary nuclear protein in testis (NUT) carcinoma: clinical, molecular characteristics, and treatment strategies. BMC Cancer. (2025) 25(1):196. 10.1186/s12885-025-13593-3 39905380 PMC11792297

[7] Flaadt T Lemelle L Abele M Virgone C Ben-Ami T Kachanov D. NUT Carcinoma in children and adolescents: an analysis of the European cooperative study group on pediatric rare tumors (EXPeRT). Lung Cancer. (2025) 201:108449. 10.1016/j.lungcan.2024.108449 39999637

[8] Sholl LM Nishino M Pokharel S Mino-Kenudson M French CA Janne PA. Primary pulmonary NUT midline carcinoma: clinical, radiographic, and pathologic characterizations. J Thorac Oncol. (2015) 10(6):951–9. 10.1097/JTO.0000000000000545 26001144 PMC4443847

[9] Ali H Bajwa S Prabhakaran SY Narra SA Ogedegbe OJ Mohsin F. NUT Carcinoma: survival outcomes and epidemiology. J Clin Oncol. (2025) 43(16_suppl):e22540. 10.1200/JCO.2025.43.16_suppl.e22540

[10] French CA. NUT Carcinoma: clinicopathologic features, pathogenesis, and treatment. Pathol Int. (2018) 68(11):583–95. 10.1111/pin.12727 30362654

[11] Haack H Johnson LA Fry CJ Crosby K Polakiewicz RD Stelow EB. Diagnosis of NUT midline carcinoma using a NUT-specific monoclonal antibody. Am J Surg Pathol. (2009) 33(7):984–91. 10.1097/PAS.0b013e318198d666 19363441 PMC2783402

[12] Kim JJ Walton SA Mahadevan NR Haradon J Paoloni F Paik PK. Molecular characterization of NUT carcinoma: a report from the NUT carcinoma registry. Clin Cancer Res. (2025) 31(18):3922–31. 10.1158/1078-0432.CCR-25-1071 40704901 PMC12373430

[13] Walton SA Kim JJ Paoloni F Liew JW Haradon D Haradon J. Presenting features and causes of diagnostic delays: a report from the NUT carcinoma registry. JCO Precis Oncol. (2025) 9:e2500526. 10.1200/PO-25-00526 41348986 PMC12695031

[14] Schlemmer AJ Gorkiewicz G Uggowitzer MM Salamon S Jost P Talakic E. 18 F-FDG PET/CT in NUT carcinoma of the thorax. Clin Nucl Med. (2025) 50(11):1062–3. 10.1097/RLU.0000000000005872 40302130

[15] Huang W Zhang Y Yang Q Gao G Qiu Y Li L. Clinical imaging of primary pulmonary nucleoprotein of the testis carcinoma. Front Med (Lausanne). (2022) 9:1083206. 10.3389/fmed.2022.1083206 36687409 PMC9845940

[16] Pelosi G Cannone M Balladore E Rahal D Bossi P Novellis P. Spindle cell nuclear in testis carcinoma of the lung: a challenging tumor. J Thorac Oncol. (2019) 14(2):311–3. 10.1016/j.jtho.2018.09.007 30476575

[17] Baras AS Naidoo J Hann CL Illei PB Reninger CW Lauring J. Rediagnosis of lung cancer as NUT midline carcinoma based on clues from tumor genomic profiling. J Natl Compr Canc Netw. (2018) 16(5):467–72. 10.6004/jnccn.2017.7203 29752320

[18] Pasricha S Jajodia A Sharma A Bansal D Batra U Gupta G. Primary pulmonary NUT midline carcinoma: an elusive and a rare diagnostic entity. J Cancer Res Ther. (2023) 19(3):816–8. 10.4103/jcrt.jcrt_1168_21 37470617

[19] Martins B Guimarães S Araújo D. Primary pulmonary NUT carcinoma: an aggressive and rare tumor. Arch Bronconeumol. (2025) 61(1):52–4. 10.1016/j.arbres.2024.09.012 39438202

[20] Numakura S Saito K Motoi N Mori T Saito Y Yokote F. P63-negative pulmonary NUT carcinoma arising in the elderly: a case report. Diagn Pathol. (2020) 15(1):134. 10.1186/s13000-020-01053-4 33176817 PMC7657348

[21] Maruyama N Hikiishi A Suginaka M Furukawa K Ogawa K Nakamura N. Nuclear protein in testis carcinoma of the thorax. Intern Med. (2018) 57(21):3169–73. 10.2169/internalmedicine.0434-17 29877266 PMC6262720

[22] Wu SJ Kim JJ Huang Y Durall RT Becker S Canty S. Novel BRD2::NUTM1 fusion in NUT carcinoma with exceptional response to chemotherapy: a case report. JTO Clin Res Rep. (2024) 5(1):100625. 10.1016/j.jtocrr.2023.100625 38287941 PMC10823067

[23] Chang AI Kim TS Han J Kim TJ Choi JY. NUT Midline carcinoma of the lung: computed tomography findings in 10 patients. J Comput Assist Tomogr. (2021) 45(2):330–6. 10.1097/RCT.0000000000001133 33661151

[24] Li Y Zhu Z Peng L Jin Z Sun L Song B. NUT Midline carcinoma in a young pregnant female: a case report. World J Surg Oncol. (2020) 18(1):290. 10.1186/s12957-020-02063-8 33160369 PMC7648955

[25] Sakaguchi T Furuya N Ito K Hida N Morikawa K Komase Y. Lung nuclear protein in testis carcinoma in an elderly Korean woman: a case report with cytohistological analysis. Thorac Cancer. (2020) 11(6):1724–7. 10.1111/1759-7714.13429 32319714 PMC7262936

[26] Sun Y Bian J Wang L Zhang T. Etoposide and cisplatin in combination with anlotinib for lung NUT carcinoma: a case report. Front Oncol. (2025) 15:1632133. 10.3389/fonc.2025.1632133 41040524 PMC12483845

[27] Argyropoulos KV Lin LH Moreira AL Krock B Simsir A Brandler TC. Cytological features of NUT-carcinoma harbouring an NSD3-NUTM1 fusion. Cytopathology. (2022) 33(4):540–3. 10.1111/cyt.13120 35325484

[28] Xie M Fu X Wang W. Clinicopathological and molecular characterizations of pulmonary NUT midline carcinoma. Cancer Med. (2021) 10(17):5757–64. 10.1002/cam4.4096 34409758 PMC8419746

[29] Kloker LD Sidiras M Flaadt T Brecht IB Deinzer CKW Groß T. Clinical management of NUT carcinoma (NC) in Germany: analysis of survival, therapy response, tumor markers and tumor genome sequencing in 35 adult patients. Lung Cancer. (2024) 189:107496. 10.1016/j.lungcan.2024.107496 38301600

[30] Shan G Zeng D Huang W Yao G. An early-stage nuclear protein in testis carcinoma of the lung in an older woman. Interdiscip Cardiovasc Thorac Surg. (2026) 41(1):ivaf278. 10.1093/icvts/ivaf278 41339294 PMC12774462

[31] Lee T Choi S Han J Choi YL Lee K. Abrupt dyskeratotic and squamoid cells in poorly differentiated carcinoma: case study of two thoracic NUT midline carcinomas with cytohistologic correlation. J Pathol Transl Med. (2018) 52(5):349–53. 10.4132/jptm.2018.07.16 30056636 PMC6166013

[32] Matsuura H Makimoto G Oda N Ninomiya K Higo H Fujii M. A prompt diagnosis and treatment of a case of nuclear protein of the testis carcinoma characterized by a bronchial lesion and high Serum alpha-fetoprotein level following genomic testing. Intern Med. (2024) 63(19):2655–60. 10.2169/internalmedicine.2938-23 38403772 PMC11518595

[33] Labiano T Fuentes M Rodriguez Y Cascante Rodrigo JA Lozano MD Dot T. A case report of undifferentiated NUT carcinoma of lung diagnosed by EBUS-FNA: if you hear hoofbeats do not assume they are horses. Diagn Cytopathol. (2025):1–16. 10.1002/dc.70054 41277582

[34] Ballenberger M Vojnic M Indaram M Machnicki S Harshan M Novoselac AV. A 33-year-old man with chest pain. Chest. (2022) 161(1):e43–43e49. 10.1016/j.chest.2021.08.069 35000716

[35] Zhang Y Zhang Q Hao Y Luo J Piao Y Wang W. International guidelines on the diagnosis and treatment of NUT carcinoma. Innovation (Camb). (2026) 7(1):101068. 10.1016/j.xinn.2025.101068 41737331 PMC12925906

[36] Durall RT Huang J Wojenski L Huang Y Gokhale PC Leeper BA. The BRD4-NUT fusion alone drives malignant transformation of NUT carcinoma. Cancer Res. (2023) 83(23):3846–60. 10.1158/0008-5472.CAN-23-2545 37819236 PMC10690098

[37] Zheng D Elnegiry AA Luo C Bendahou MA Xie L Bell D. Brd4::Nutm1 fusion gene initiates NUT carcinoma *in vivo*. Life Sci Alliance. (2024) 7(7):e202402602. 10.26508/lsa.202402602 38724194 PMC11082452

[38] Eagen KP French CA. Supercharging BRD4 with NUT in carcinoma. Oncogene. (2021) 40(8):1396–408. 10.1038/s41388-020-01625-0 33452461 PMC7914217

[39] Yang G Liu R Yang L Yang X Tang X Mao H. Pulmonary NUT carcinoma, an elusive and refractory entity, shows transient response to chemotherapeutics and PD-1 inhibitor: a case report and literature review. Front Immunol. (2025) 16:1497124. 10.3389/fimmu.2025.1497124 40134436 PMC11932979

[40] Abdulwali SK El-Sibai AM Ali SS Alaklabi AM Abdalla HM Sabbah AN. Predictive and prognostic factors in NUT carcinoma: a systematic review and individual patient data meta-analysis. Cancer Res. (2024) 84(6 suppl):3826. 10.1158/1538-7445.AM2024-3826

[41] Luo J Bishop JA Dubois SG Hanna GJ Sholl LM Stelow EB. Hiding in plain sight: NUT carcinoma is an unrecognized subtype of squamous cell carcinoma of the lungs and head and neck. Nat Rev Clin Oncol. (2025) 22(4):292–306. 10.1038/s41571-025-00988-5 39900969 PMC12077380

[42] Ye Z Li X Zhang M Xie F Luo X Deng C. Complete response to BET inhibitor in primary pulmonary NUT carcinoma with single-cell sequencing-based analysis: a case report. JTO Clin Res Rep. (2025) 6(10):100885. 10.1016/j.jtocrr.2025.100885 40926997 PMC12414819

[43] Liu H Gong W Ji Y Xu T Zhang C. 68Ga-FAP-2286 PET/CT imaging of nuclear protein of the testis midline carcinoma in the lung. Clin Nucl Med. (2025) 50(12):1176–7. 10.1097/RLU.0000000000006020 40474365

[44] Yuan J Xu Z Diagnosis GY. Treatment and prognosis of primary pulmonary NUT carcinoma: a literature review. Curr Oncol. (2022) 29(10):6807–15. 10.3390/curroncol29100536 36290813 PMC9600367

[45] Roden AC. Molecularly defined thoracic neoplasms. Adv Anat Pathol. (2024) 31(5):303–17. 10.1097/PAP.0000000000000439 38501690

[46] Lantuejoul S Pissaloux D Ferretti GR McLeer A. NUT Carcinoma of the lung. Semin Diagn Pathol. (2021) 38(5):72–82. 10.1053/j.semdp.2021.06.005 34176698

