# Supplementary material for: Systematic review of PET imaging features and clinical applications in pulmonary NUT carcinoma
Source: Front Radiol. 2026 Aug 28;6:1933175. doi: 10.3389/fradi.2026.1933175 (PMC13562259; doi:10.3389/fradi.2026.1933175)
Supplement: Supplementary file 1 [file Table1.docx]

**Supplementary table 1. Quality Assessment of the Included Retrospective Cohort Study (Newcastle-Ottawa Scale)**

| First Author (Year) | Selection | Comparability | Outcome | Total NOS Score | Risk of Bias |
| --- | --- | --- | --- | --- | --- |
| Kloker LD et al. (2024) | ★★★ | — | ★★★ | 5★ | Moderate |

Note: The maximum NOS score is 9★. Selection items include representativeness of the exposed cohort, ascertainment of exposure, and demonstration that the outcome of interest was not present at the start of study (3★). Comparability was not applicable owing to the absence of a control group. Outcome items include assessment of outcome, sufficient duration of follow-up, and adequacy of follow-up (3★).

**Supplementary table 2. Quality Assessment of the Included Case Series (JBI Checklist for Case Series)**

| First Author (Year) | Q1 | Q2 | Q3 | Q4 | Q5 | Q6 | Q7 | Q8 | Q9 | Q10 | Compliance Rate | Risk of Bias |
| --- | --- | --- | --- | --- | --- | --- | --- | --- | --- | --- | --- | --- |
| Xie M et al. (2021) | Y | Y | Y | Y | Y | Y | Y | Y | Y | Y | 100% | Low |
| Sholl LM et al. (2015) | Y | Y | Y | Y | Y | Y | Y | Y | Y | Y | 100% | Low |
| Huang W et al. (2023) | Y | Y | Y | Y | Y | Y | Y | Y | Y | Y | 100% | Low |
| Chang AI et al. (2021) | Y | Y | Y | Y | Y | Y | Y | Y | Y | Y | 100% | Low |
| Qu J et al. (2025) | Y | Y | Y | Y | Y | Y | Y | Y | Y | Y | 100% | Low |

Note: JBI Checklist for Case Series items (Q1–Q10): Q1 = Were the criteria for inclusion in the case series clearly described? Q2 = Was the condition measured using standard criteria? Q3 = Were valid methods used for the identification of the condition? Q4 = Were the cases included consecutively? Q5 = Were the cases included completely? Q6 = Was the demographic information of the participants clearly reported? Q7 = Was the clinical information of the participants clearly reported? Q8 = Were the outcomes or follow-up results of cases clearly reported? Q9 = Was the demographic information of the presenting site(s)/clinic(s) clearly reported? Q10 = Was the statistical analysis appropriate? Y = Yes.

**Supplementary table 3. Quality Assessment of the Included Case Reports (JBI Checklist for Case Reports)**

| First Author (Year) | Q1 | Q2 | Q3 | Q4 | Q5 | Q6 | Q7 | Q8 | Compliance Rate | Risk of Bias |
| --- | --- | --- | --- | --- | --- | --- | --- | --- | --- | --- |
| Pelosi G et al. (2016) | Y | Y | Y | Y | Y | Y | Y | Y | 100% | Low |
| Wu SJ et al. (2023) | Y | Y | Y | Y | Y | Y | Y | Y | 100% | Low |
| Baras AS et al. (2018) | Y | Y | Y | Y | Y | Y | Y | Y | 100% | Low |
| Shan G et al. (2025) | Y | Y | Y | Y | Y | Y | Y | Y | 100% | Low |
| Cho HJ et al. (2020) | Y | Y | Y | Y | Y | Y | Y | Y | 100% | Low |
| Sun Y et al. (2025) | Y | Y | Y | Y | Y | Y | Y | Y | 100% | Low |
| Maruyama N et al. (2018) | Y | Y | Y | Y | Y | Y | Y | Y | 100% | Low |
| Pasricha S et al. (2023) | Y | Y | Y | Y | Y | Y | Y | Y | 100% | Low |
| Argyropoulos KV et al. (2022) | Y | Y | Y | Y | Y | Y | Y | Y | 100% | Low |
| Matsuura H et al. (2024) | Y | Y | Y | Y | Y | Y | Y | Y | 100% | Low |
| Lee T et al. (2018) | Y | Y | Y | Y | Y | Y | Y | Y | 100% | Low |
| Joel S et al. (2020) | Y | Y | Y | Y | Y | Y | Y | Y | 100% | Low |
| Numakura S et al. (2020) | Y | Y | Y | Y | Y | Y | Y | Y | 100% | Low |
| Schlemmer AJ et al. (2025) | Y | Y | Y | Y | Y | Y | Y | Y | 100% | Low |
| Ballenberger M et al. (2022) | Y | Y | Y | Y | Y | Y | Y | Y | 100% | Low |
| Labiano T et al. (2025) | Y | Y | Y | Y | Y | Y | Y | Y | 100% | Low |
| Martins B et al. (2024) | Y | Y | Y | Y | Y | Y | Y | Y | 100% | Low |

Note: JBI Checklist for Case Reports items (Q1–Q8): Q1 = Were the patient's demographic characteristics clearly described? Q2 = Was the patient's history described in a clear chronological order? Q3 = Was the patient's current clinical condition clearly described? Q4 = Were the diagnostic or assessment methods clearly described? Q5 = Were the intervention(s) or treatment procedure(s) clearly described? Q6 = Was the post-intervention clinical condition clearly described? Q7 = Were adverse events or unanticipated events identified and described? Q8 = Does the case report provide takeaway lessons or experience? Y = Yes.
