# Supplementary material for: Systematic review of PET imaging features and clinical applications in pulmonary NUT carcinoma
Source: Front Radiol. 2026 Aug 28;6:1933175. doi: 10.3389/fradi.2026.1933175 (PMC13562259; doi:10.3389/fradi.2026.1933175)
Supplement: Supplementary file 2 [file Table2.docx]

| **Section and Topic** | **Item #** | **Checklist item** | **Location where item is reported** |
| --- | --- | --- | --- |
| **TITLE** | | |  |
| Title | 1 | Identify the report as a systematic review. | Title: Systematic Review of PET Imaging Features and Clinical Applications in Pulmonary NUT Carcinoma |
| **ABSTRACT** | | |  |
| Abstract | 2 | See the PRISMA 2020 for Abstracts checklist. | See PRISMA 2020 for Abstracts checklist. The structured abstract includes: Purpose, Methods, Results, and Conclusions. |
| **INTRODUCTION** | | |  |
| Rationale | 3 | Describe the rationale for the review in the context of existing knowledge. | Introduction, Paragraphs 17–20 |
| Objectives | 4 | Provide an explicit statement of the objective(s) or question(s) the review addresses. | Introduction, Paragraph 21 |
| **METHODS** | | |  |
| Eligibility criteria | 5 | Specify the inclusion and exclusion criteria for the review and how studies were grouped for the syntheses. | Methods, Section 2.2 (Eligibility Criteria), Paragraphs 32–44 |
| Information sources | 6 | Specify all databases, registers, websites, organisations, reference lists and other sources searched or consulted to identify studies. Specify the date when each source was last searched or consulted. | Methods, Section 2.1 (Search Strategy), Paragraphs 24–25; databases (PubMed, Embase, Cochrane Library, Web of Science), grey literature (ProQuest Dissertations, ASCO/ESMO/IASLC conference abstracts), and reference lists |
| Search strategy | 7 | Present the full search strategies for all databases, registers and websites, including any filters and limits used. | Methods, Section 2.1 (Search Strategy), Paragraphs 25–29; PubMed search strategy presented; adapted for other databases |
| Selection process | 8 | Specify the methods used to decide whether a study met the inclusion criteria of the review, including how many reviewers screened each record and each report retrieved, whether they worked independently, and if applicable, details of automation tools used in the process. | Methods, Section 2.3 (Study Selection and Data Extraction), Paragraphs 45–46 |
| Data collection process | 9 | Specify the methods used to collect data from reports, including how many reviewers collected data from each report, whether they worked independently, any processes for obtaining or confirming data from study investigators, and if applicable, details of automation tools used in the process. | Methods, Section 2.3 (Study Selection and Data Extraction), Paragraphs 47–48 |
| Data items | 10a | List and define all outcomes for which data were sought. Specify whether all results that were compatible with each outcome domain in each study were sought (e.g. for all measures, time points, analyses), and if not, the methods used to decide which results to collect. | Methods, Section 2.3 (Study Selection and Data Extraction), Paragraph 47; Outcomes listed in Section 2.2 (Inclusion Criteria), Paragraph 36 |
|  | 10b | List and define all other variables for which data were sought (e.g. participant and intervention characteristics, funding sources). Describe any assumptions made about any missing or unclear information. | Methods, Section 2.3 (Study Selection and Data Extraction), Paragraph 47; Table 1 (study and patient characteristics) |
| Study risk of bias assessment | 11 | Specify the methods used to assess risk of bias in the included studies, including details of the tool(s) used, how many reviewers assessed each study and whether they worked independently, and if applicable, details of automation tools used in the process. | Methods, Section 2.4 (Quality Assessment), Paragraphs 49–50; Supplementary Tables S1–S3 |
| Effect measures | 12 | Specify for each outcome the effect measure(s) (e.g. risk ratio, mean difference) used in the synthesis or presentation of results. | Methods, Section 2.5 (Data Synthesis), Paragraphs 51–52. No meta-analysis was performed; structured qualitative synthesis was conducted. |
| Synthesis methods | 13a | Describe the processes used to decide which studies were eligible for each synthesis (e.g. tabulating the study intervention characteristics and comparing against the planned groups for each synthesis (item #5)). | Methods, Section 2.5 (Data Synthesis), Paragraphs 51–52 |
|  | 13b | Describe any methods required to prepare the data for presentation or synthesis, such as handling of missing summary statistics, or data conversions. | Methods, Section 2.5 (Data Synthesis), Paragraph 52; continuous variables with reporting rates >80% summarised using ranges and medians |
|  | 13c | Describe any methods used to tabulate or visually display results of individual studies and syntheses. | Results, Tables 1–5 and Figure 1; structured tabulation and qualitative description |
|  | 13d | Describe any methods used to synthesize results and provide a rationale for the choice(s). If meta-analysis was performed, describe the model(s), method(s) to identify the presence and extent of statistical heterogeneity, and software package(s) used. | Methods, Section 2.5 (Data Synthesis), Paragraphs 51–52; structured qualitative synthesis without meta-analysis was chosen due to substantial inter-study heterogeneity and the predominance of case reports/series |
|  | 13e | Describe any methods used to explore possible causes of heterogeneity among study results (e.g. subgroup analysis, meta-regression). | No subgroup analysis or meta-regression was conducted due to insufficient number of comparable studies |
|  | 13f | Describe any sensitivity analyses conducted to assess robustness of the synthesized results. | Methods, Section 2.8 (Sensitivity Analysis), Paragraph 58; Results, Section 3.7 (Sensitivity Analyses), Paragraph 89 |
| Reporting bias assessment | 14 | Describe any methods used to assess risk of bias due to missing results in a synthesis (arising from reporting biases). | Methods, Section 2.6 (Publication Bias Assessment), Paragraphs 53–54; qualitative assessment only due to limited statistical power |
| Certainty assessment | 15 | Describe any methods used to assess certainty (or confidence) in the body of evidence for an outcome. | Methods, Section 2.7 (GRADE Evidence Rating), Paragraphs 55–56; Results, Section 3.7 (GRADE Evidence Profile), Paragraph 88 |
| **RESULTS** | | |  |
| Study selection | 16a | Describe the results of the search and selection process, from the number of records identified in the search to the number of studies included in the review, ideally using a flow diagram. | Results, Section 3.1 (Study Selection), Paragraphs 60–61; Figure 1 (PRISMA 2020 flow diagram) |
|  | 16b | Cite studies that might appear to meet the inclusion criteria, but which were excluded, and explain why they were excluded. | Not reported in detail |
| Study characteristics | 17 | Cite each included study and present its characteristics. | Results, Section 3.2 (Characteristics of Included Studies), Paragraphs 63–64; Table 1 |
| Risk of bias in studies | 18 | Present assessments of risk of bias for each included study. | Results, Section 3.7 (Quality Assessment), Paragraphs 85–86; Table 5; Supplementary Tables S1–S3 |
| Results of individual studies | 19 | For all outcomes, present, for each study: (a) summary statistics for each group (where appropriate) and (b) an effect estimate and its precision (e.g. confidence/credible interval), ideally using structured tables or plots. | Results, Sections 3.3–3.6; Tables 2–4 and supplementary tables |
| Results of syntheses | 20a | For each synthesis, briefly summarise the characteristics and risk of bias among contributing studies. | Results, Sections 3.3–3.7; Tables 1–5; narrative summaries of study characteristics and risk of bias presented for each thematic synthesis |
|  | 20b | Present results of all statistical syntheses conducted. If meta-analysis was done, present for each the summary estimate and its precision (e.g. confidence/credible interval) and measures of statistical heterogeneity. If comparing groups, describe the direction of the effect. | No statistical syntheses (meta-analyses) were conducted. Results are presented as structured qualitative synthesis in Sections 3.3–3.6 and Tables 2–4. |
|  | 20c | Present results of all investigations of possible causes of heterogeneity among study results. | No formal investigations of heterogeneity (subgroup analysis or meta-regression) were conducted. |
|  | 20d | Present results of all sensitivity analyses conducted to assess the robustness of the synthesized results. | Results, Section 3.7, Paragraph 89; sensitivity analyses showed no substantive impact on conclusions. |
| Reporting biases | 21 | Present assessments of risk of bias due to missing results (arising from reporting biases) for each synthesis assessed. | Results, Section 3.7 (Heterogeneity and Evidence Profile), Paragraph 87; qualitative assessment of publication bias |
| Certainty of evidence | 22 | Present assessments of certainty (or confidence) in the body of evidence for each outcome assessed. | Results, Section 3.7 (GRADE Evidence Profile), Paragraph 88; GRADE evidence ratings presented for each outcome domain |
| **DISCUSSION** | | |  |
| Discussion | 23a | Provide a general interpretation of the results in the context of other evidence. | Discussion, Section 4.1 (Principal Findings and Academic Positioning), Paragraphs 90–93 |
|  | 23b | Discuss any limitations of the evidence included in the review. | Discussion, Section 4.5 (Limitations and Future Directions), Paragraphs 105–107 |
|  | 23c | Discuss any limitations of the review processes used. | Discussion, Section 4.5 (Limitations and Future Directions), Paragraphs 105–107 |
|  | 23d | Discuss implications of the results for practice, policy, and future research. | Discussion, Section 4.6 (Clinical Implications and Conclusions), Paragraphs 108–110 |
| **OTHER INFORMATION** | | |  |
| Registration and protocol | 24a | Provide registration information for the review, including register name and registration number, or state that the review was not registered. | Abstract, Paragraph 9; Methods, Paragraph 23; Ethics approval, Paragraph 127. PROSPERO registration: CRD420261292692. |
|  | 24b | Indicate where the review protocol can be accessed, or state that a protocol was not prepared. | Not reported (no published protocol was prepared) |
|  | 24c | Describe and explain any amendments to information provided at registration or in the protocol. | Not applicable (no amendments to registration or protocol information) |
| Support | 25 | Describe sources of financial or non-financial support for the review, and the role of the funders or sponsors in the review. | Statements and Declarations, Paragraphs 116–117 (Funding) |
| Competing interests | 26 | Declare any competing interests of review authors. | Statements and Declarations, Paragraphs 118–119 (Competing Interests) |
| Availability of data, code and other materials | 27 | Report which of the following are publicly available and where they can be found: template data collection forms; data extracted from included studies; data used for all analyses; analytic code; any other materials used in the review. | Data extracted from included studies and data used for all analyses are reported in the article and supplementary materials (Supplementary Tables S1–S3). Template data collection forms, analytic code, and other materials are not publicly available. |

*From:*  Page MJ, McKenzie JE, Bossuyt PM, Boutron I, Hoffmann TC, Mulrow CD, et al. The PRISMA 2020 statement: an updated guideline for reporting systematic reviews. BMJ 2021;372:n71. doi: 10.1136/bmj.n71
